# Age-Related Changes on CD40 Promotor Methylation and Immune Gene Expressions in Thymus of Chicken

**DOI:** 10.3389/fimmu.2018.02731

**Published:** 2018-11-21

**Authors:** Yulong Li, Xinyu Lei, Hong Lu, Wei Guo, Shengru Wu, Zhenchen Yin, Qingzhu Sun, Xiaojun Yang

**Affiliations:** College of Animal Science and Technology, Northwest A&F University, Xi'an, China

**Keywords:** chicken, thymus immunity, age, *CD40*, DNA methylation

## Abstract

One hundred and twenty one-day-old breeder cocks, included 15 cages of 8 birds each, were fed to learn the aging's effect on chicken's thymus immunity. At 2 (2-W) and 40 (40-W) weeks of age, one chicken each cage was randomly chosen and slaughtered to get the thymus sample. The results showed that thymus weight and morphology of 40-W group were far different from that of 2-W group, and exhibited a property of degeneration. Considering this phenotype variance, we analyzed the thymus' transcriptome to investigate the molecular mechanism that had been implicated in this phenotype diversity with age. Pearson correlation coefficients and principal component analysis indicated that two major populations corresponding to 40-W and 2-W group were identified, and 1949 differentially expressed genes (DEGs, 1722 up-regulated and 127 down-regulated) were obtained. Results of GO and KEGG pathway enrichment found that 4 significantly enriched KEGG pathways (Cytokine-cytokine receptor interaction, Intestinal immune network for IgA production, Toll-like receptor signaling pathway, AGE-RAGE signaling pathway in diabetic complications) related to immunoregulation were screened between 40-W and 2-W group. These results confirmed that thymus immunity of chickens had a strong age-related correlation. DEGs related to these 4 enriched KEGG pathways were suppressed in the thymus of 2-W group, this indicated that thymus immunity of 2-weeks-age chick was down-regulated. CD40 is involved in 3 of the 4 significantly enriched pathways, and it is critical for thymus immune-regulation. CD40 promoter methylation level of 2-W group was higher than that of 40-W group, it is consistent with the transcriptional differences of the gene. Our study concluded that thymus immunity of chicken was varied with age. Compared to the 40-W group, thymus immunity of 2-W group was down-regulated, and in a status of hypo-activation on the whole, and these effects might be related to CD40 suppression induced by promoter hyper-methylation of the gene.

## Introduction

As central immunological organs, thymus is an essential organ for the generation of the adaptive immune system (1), as well as an organ where T cells grow, differentiation and to be mature finally (2). After differentiation from bone marrow, T lymphocytes will migrate into thymus with the assistance of chemokines, and finally become mature T lymphocytes (3–5). Meanwhile, Thymus itself can also generate a large pool of immature thymocyte progenitors, and further differentiate into CD8αα^+^ intraepithelial lymphocytes, invariant natural killer T cells, natural Foxp3^+^ regulatory T cells, and natural T helper cells (6). These thymic immunocytes will collaborate together to be responsive to foreign antigens and tolerant to self, and further modulate the health of adaptive immune system (6–8). After long-term co-evolution, thymus could successfully deal with a great variety of pathogens and passed through antigen-rich environments. Foreign antigens, mostly coming from intestinal microbiota, can be incepted by thymic antigen presenting cells, and involved in antigen processing and presentation. And then, antigen processing and presentation will be implicated in differentiation and repertoire selection of T cell in thymus (8, 9). This means that pathogen associated molecule patterns (PAMP) from microbiota are critical for induction and development of thymus.

Across the lifespan, thymus has distinct developmental and morphological characteristic at different ages. As to mammals, previous research found that thymus grew and developed rapidly after birth. And then, the slowdown and deterioration of the thymus would begin shortly after teenage years (10). As to humans, who will experience a relatively long lifespan, the immune function of lymphocyte from thymus shows high diversity in different life stages (11, 12). For bird, as well, the functional status of thymus are changing in different degrees with age growth. Chick at 2-weeks-age is young, and its thymus is still in the developing stage. When it comes to 40-weeks-age, thymus of adult chicken maybe in the status of degeneration as adult mammals. To a large extent, the functional status of thymus are determined by the expressed genes, so we analyze the genes expression at the level of transcription to learn the thymus immunity differences with age (2 and 40 weeks age).

DNA methylation, as an important kind of epigenetic modification, could modulate genes expression at the transcriptional level (13, 14). Previous researches found that DNA methylation had a charicteristic of high in stability, and status of DNA methylation could be maintained for a long time. DNA (de)methylation was a very time-intensive process (more than 1 week), and so, DNA methylation could stabilize the status of genes transcription (activated or suppressed) (15–17). Genes transcription and functional status of thymus could also be last out a long period. We hypothesized that genes transcriptional changes of thymus with age were related to their differential DNA methylation. So, we analyzed the genes transcriptome, as well as promoter methylation of core regulator in thymus to learn the thymus immunity at different developmental stages.

## Materials and methods

### Animals

The animal protocol for our study was approved strictly by the Animal Care and Use Committee of the College of Animal Science and Technology of the Northwest A&F University (Shaanxi, China). 120 one-day-old Avein chickens were purchased from Poultry Breeding co., LTD of Beijing (Beijing, China), and fed in 15 cages, 8 birds each cage. All the chickens had a corn-soybean meal diet (Table S1), and drank water freely. At 2 (2-W) and 40 (40-W) weeks of age, one chicken each cage was randomly chosen and slaughtered. The second thymus on the right was collected to analyze the morphology and weight of thymus. Then we quickly collected the thymus on the left, and frozen immediately in liquid nitrogen, and stored at −80°C for RNA-seq, qRT-PCR and BSP analysis.

### RNA-seq library preparation and quality control analysis

Total RNA was extracted from thymus by using TRIzol reagent (Takara) following manufacturer's instructions, and then treated with DNase I to remove the genomic DNA. The purity and quantity of total RNA were analyzed using Nanodrop equipment. Messenger RNA (mRNA) was isolated using magnetic beads with Oligo (dT) and fragmented into short fragments with the fragmentation buffer. Then cDNA was synthesized using the mRNA fragments as templates, after that, the cDNA was purified and resolved with EB buffer for end reparation and single nucleotide A (adenine) addition. After agarose gel electrophoresis, the suitable fragments were selected for the PCR amplification as templates. Quality of the sample library was checked on a 2100 Bioanalyser (Agilent, Waldbroon, Germany), and the related indexes include clean reads number, base percentage composition along reads and so on all had been calculated and assessed. At last, the sample library was sequenced using Illumina HiSeq^TM^ 2000.

### Alignment of clean reads to the reference sequences

Then, we aligned the pair-end clean raw reads to gallus reference genome (ftp.ncbi.nih.gov/genomes/Gallus_gallus/Assembledchromosomes/seq) and reference genes (/ifs1/pub/database/ftp.ncbi.nih.gov/genomes/Gallus_gallus/RNA/rna.fa.gz) by SOAP-aligner/SOAP2. No more than 5 mismatches each read are allowed in the alignment. Then, we calculated the distributions of reads on reference genes and the gene coverage distribution to assess the quality of alignment.

### Screening and significant test for differentially-expressed genes (DEGs)

The relative transcriptional level of unigene was calculated by using reads per kb per million reads (RPKM) method. We can screen DEGs between 40-W group and 2-W group on the basis of genes' RPKM (Table S2). In this study, a rigorous formula was applied to categorize different expression levels of genes. Divided each gene's RPKM of 40-W group by that of 2-W group, and we get fold change of each unigene. For further analysis, DEGs were selected where (i) the absolute values of Log2 (fold change) >1, (ii) the *p*-value (Independent sample *T*-test) was < 0.05, and (iii) the average RPKM was >0.5.

### Hierarchical cluster analysis

Hierarchical cluster analysis was performed to analyze the genes expression pattern of the DEGs on the basis of the *Z*-score values, and was performed using the Euclidean distance on the cluster 3.0 software. The *Z*-score of each thymus sample was calculated, respectively using the formula: *Z* = (x–μ)/σ, x is the RPKM of the transcript, μ and σ represent the mean and standard deviation of the transcript, respectively.

### GO and KEGG pathway enrichment analysis of DEGs

Gene Ontology (GO) is an international standard gene functional classification system to describe the properties of genes and their products, which includes three aspects: molecular function, cellular component and biological process. The Kyoto Encyclopedia of Genes and Genomes (KEGG) pathway enrichment analysis for the DEGs (18) was conducted to identify significantly enriched metabolic pathways or signal transduction pathways in DEGs comparing with the whole genome background. GO and KEGG enrichment analysis for the screened DEGs was carried out using online KOBAS 3.0 (http://kobas.cbi.pku.edu.cn/anno_iden.php) software.

### Validation the transcription of DEGs using qRT-PCR

The total RNA extracted from thymus organs of both 2-W and 40-W groups was used for fluorescent quantitative PCR analysis (qRT-PCR). The cDNA was synthesized using the PrimeScript RT reagent Kit (TaKaRa). The qRT-PCR was performed with SYBR® Premix Ex Taq TM II (TaKaRa) following the protocol provided by manufacturer on an IQ5 quantitative PCR instrument (Bio-Rad Laboratories, Hercules, CA), and programmed as follows: 95°C for 5 min; 40 cycles of 95°C for 10 s, 60°C for 30 s, 72°C for 30 s; and 72°C for 5 min. The 20 μL RT-qPCR mixture included: 10 μL of SYBR Premix Ex Taq II (2 ×), 1 μL of forward primer (10 μM), 1 μL of reverse primer (10 μM), 1 μL of template DNA, and 7 μL of ultrapure water. The reference gene, β-actin was used as an internal expression control. All samples were run in triplicate, and 2^−ΔΔ*Ct*^ method was used to calculate the transcriptional level of the selected DEGs. The primer sequences (Table S3) for the 8 DEGs (TLR1, TLR4, TLR5, CD40, AP-1, IL8, TACI, and PIGR) were designed using online NCBI/Primer-BLAST software (https://www.ncbi.nlm.nih.gov/tools/primer-blast/).

### Bisulfite sequencing PCR (BSP)

Genomic DNA was extracted from the thymus using QIAamp DNA Mini Kit (51306; Qiagen) following the manufacturer's protocol, and the extracted DNA was quantified using the NanoDrop ND-1000 spectrophotometer (Thermo Scientific). Bisulfate treatment of the DNA samples (~200 ng/sample) was performed using the EZ DNA Methylation-Gold™ Kit (A-D5005; Zymo research, USA). The modified DNA samples were diluted in 10 μL of ddH_2_O and served as templates for PCR amplification. And the BSP primers (Figure **6A**) for promoter CpG island (−848 ~ −648, 17 CpGs) of CD40 were designed using online MethPrimer software (19). The 20 uL BSP-PCR mixture contained the following: 10 μL of 2 × Premix Taq^TM^ (R004A; Takara), 1 μL of template DNA, 1 μL of forward primer (10 μM), 1 μL of reverse primer (10 μM), and 7 μL of ultrapure water. And the BSP-PCR was performed in a DNA Engine Thermal Cycler (Bio-Rad, USA) using the following program: 3 min at 95°C followed by 35 cycles of denaturation for 30 s at 95°C, annealing for 30 s at 60°C and extension for 30 s at 72°C and a final extension at 72°C for 5 min. The PCR products showed a clear band in 2% agarose gels (Figure S1) and were purified using Agarose Gel DNA Purification Kit (DV805A; Takara). Then the purified DNA fragments were subcloned using a pMD19-T Vector and transformed into chemically competent *Escherichia coli*. Fifteen DNA samples were performed in 2-W and 40-W group, respectively. And 5–7 positive clones of each sample were sequenced (BGI, Shenzhen, China). The sequencing results of BSP were statistically analyzed and plotted using an online Quantification tool for Methylation Analysis software (http://quma.cdb.riken.jp/) (20).

### Statistical analysis

Statistical analyses were analyzed by using SPSS 19.0 software. To determine the thymus differences between 40-W group and 2-W group, Student's *t*-test was adopted to analyze the data of thymus weight, genes transcription, and overall CD40 promotor methylation. And chi-square test was adopted to analyze the data of single CpG site methylation. Results were presented as the mean ± SE, and a *P*-value < 0.05 was considered statistically significant.

## Results

### Thymus weight and morphology differences between 2-W and 40-W group

**Figure 1:**
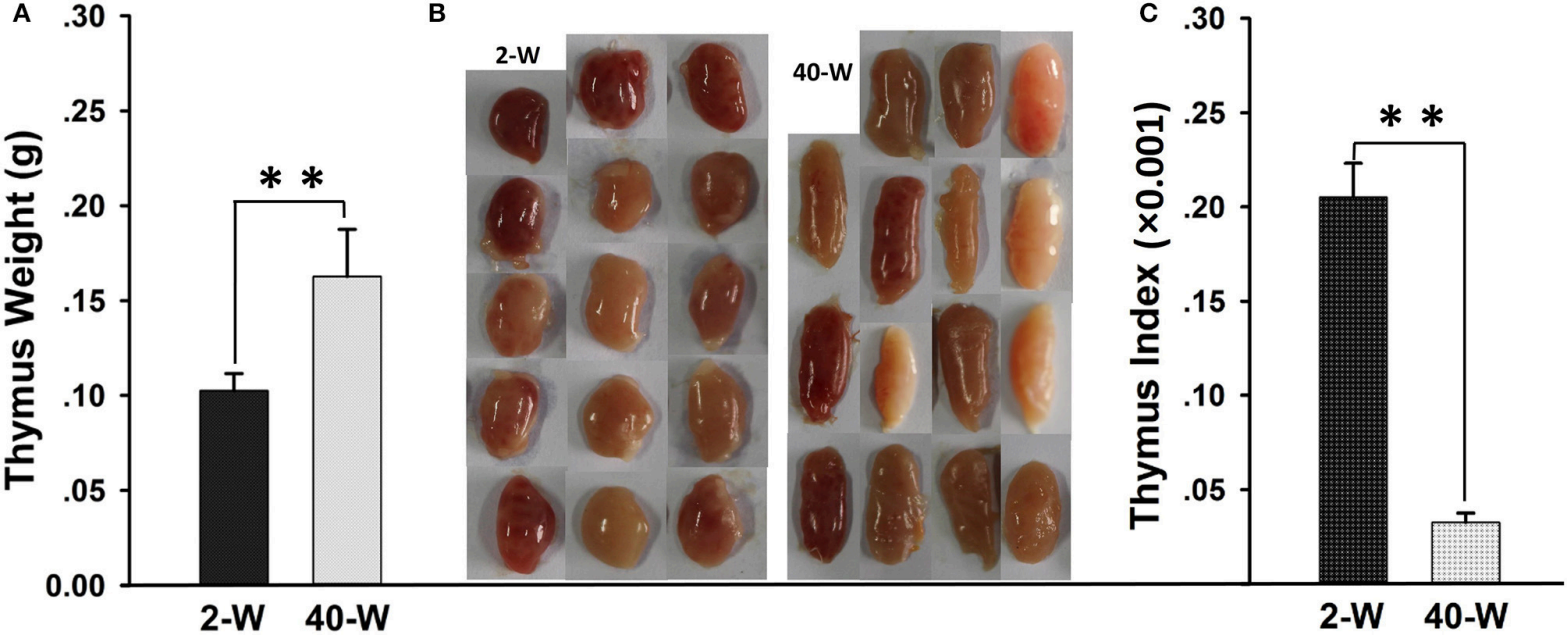
Weight and morphology of thymus. Thymus weight **(A)**, thymus index **(C)**, and thymus morphology **(B)** of 40-W group were highly different from those of 2-W group. Data shown as mean ± SD, **P < 0.01.

It showed that the thymus weight of 40-W group was highly significantly heavier (*p* < 0.01) than that of 2-W group (Figure 1A). Whereas, when it comes to thymus index, 40-W group was significantly smaller (*p* < 0.01) than that of 2-W group (Figure 1C). Compared with the 2-W group, thymus morphology of 40-week-old chickens would tend to be thinner, and much more oval and elongate (Figure 1B).

### Overview of RNA-Seq data

All the 30 thymus samples' RNA quality could meet the need of transcriptome sequencing (RNA-Seq) library construction (Table S4). And then, we assessed the quality of RNA-Seq, the related indexes including clean reads number, base percentage composition along reads, and so on. After filtration, illumina paired-end sequencing generated a total of 1,442,358,732 clean raw reads, and more than 46 million clean reads from each sample (Table S5). All 30 samples' base composition of raw reads was of high quality, adenine curve nearly be overlapped with thymine curve while guanine curve overlapped with cytosine curve, and there was no abnormal nucleotide base (Figure S2). Thirty samples' quality distribution of bases along reads was also been good, we can see that the percentage of the bases with low quality (< 20) was low (Figure S3). All these indexes indicated that these samples' reads could be used for further analyzation. Alignment statistics showed that about 70 percent of the total clean raw reads could be matched to the reference genome. When it comes to the reference gene, the percent of total matched reads was about 52 (Table S5). And then we calculated the distribution of reads on reference genes and the gene coverage distribution. The results showed that reads were evenly distributed on reference genes (Figure S4). These data suggested that the RNA-seq could well represent the expression level of unigenes.

### It showed a clear separation between 2-W and 40-W group based on genes transcriptional pattern

**Figure 2:**
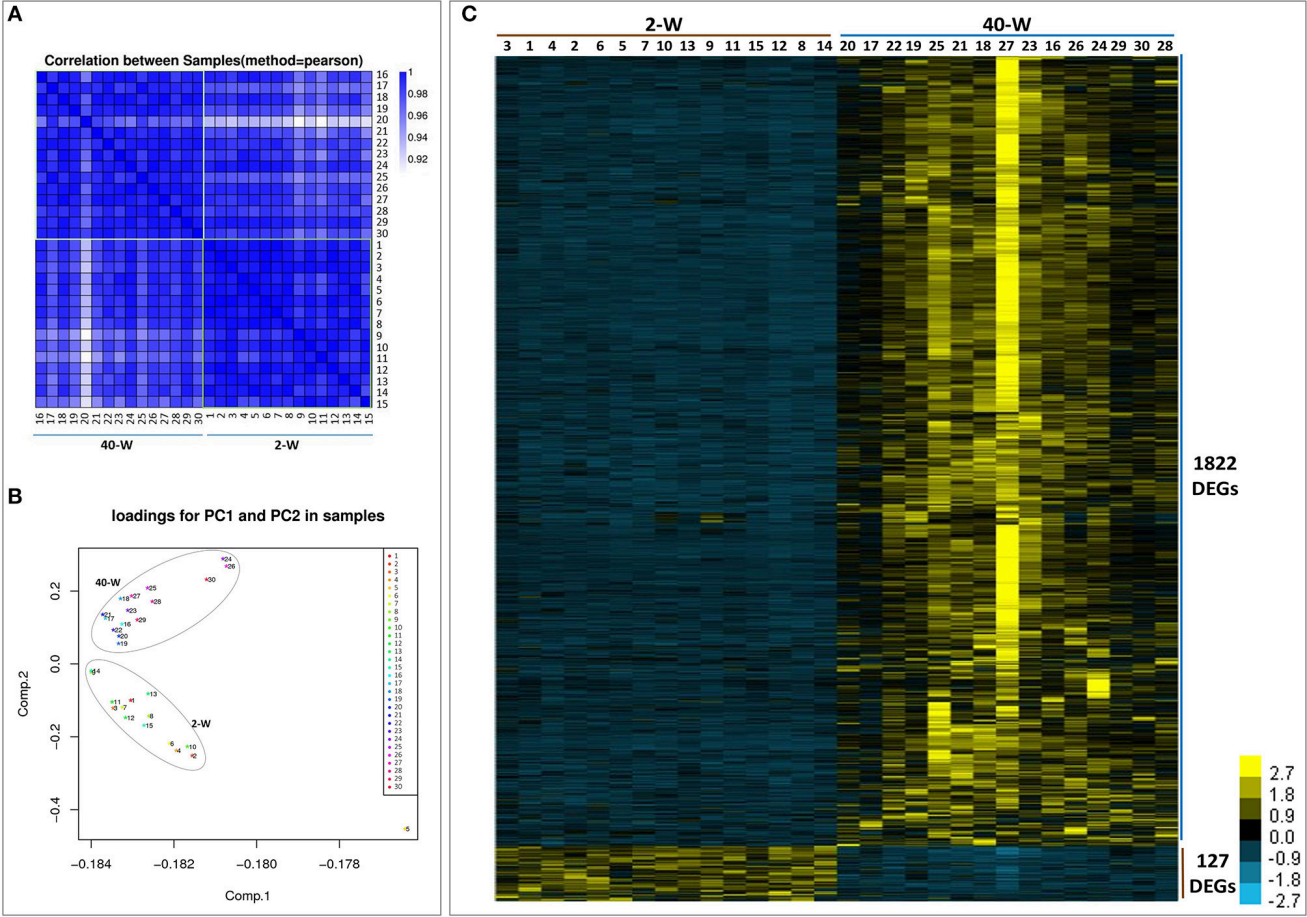
Transcriptional pattern of all the 30 samples. Results of pearson correlation coefficients **(A)** and principal component analysis **(B)** showed a clear separation between 2-W and 40-W group. And 1949 DEGs (1722 up-regulated and 127 down-regulated) were screened between 40-W and 2-W group **(C)**.

We took an unsupervised approach to partition the 30 analyzed samples into different populations based on their transcriptomic similarity. Result of Pearson correlation coefficients (PCC) showed high heterogeneity between 2-W and 40-W group, supporting the idea that genes transcription patterns in chicken thymus were significantly different between early life and adulthood stages (Figure 2A). And then, principal component analysis (PCA) was applied to further dissect the two main groups. As shown in Figure 2B, the results showed a clear separation between 2-W and 40-W group. To characterize the functional state of the two subpopulations, we screened the genes differentially expressed on the basis of RPKM. From Figure 2C, we could see that 1949 DEGs (1722 up-regulated and 127 down-regulated) were screened between 40-W and 2-W group. Hierarchical cluster analysis of DEGs was also in accordance with the results of Pearson correlation coefficients analysis and PCA, and showed distinct transcriptional patterns between 2-W and 40-W group.

### Gene ontology (GO) and KEGG pathway enrichment analysis of DEGs

**Figure 3:**
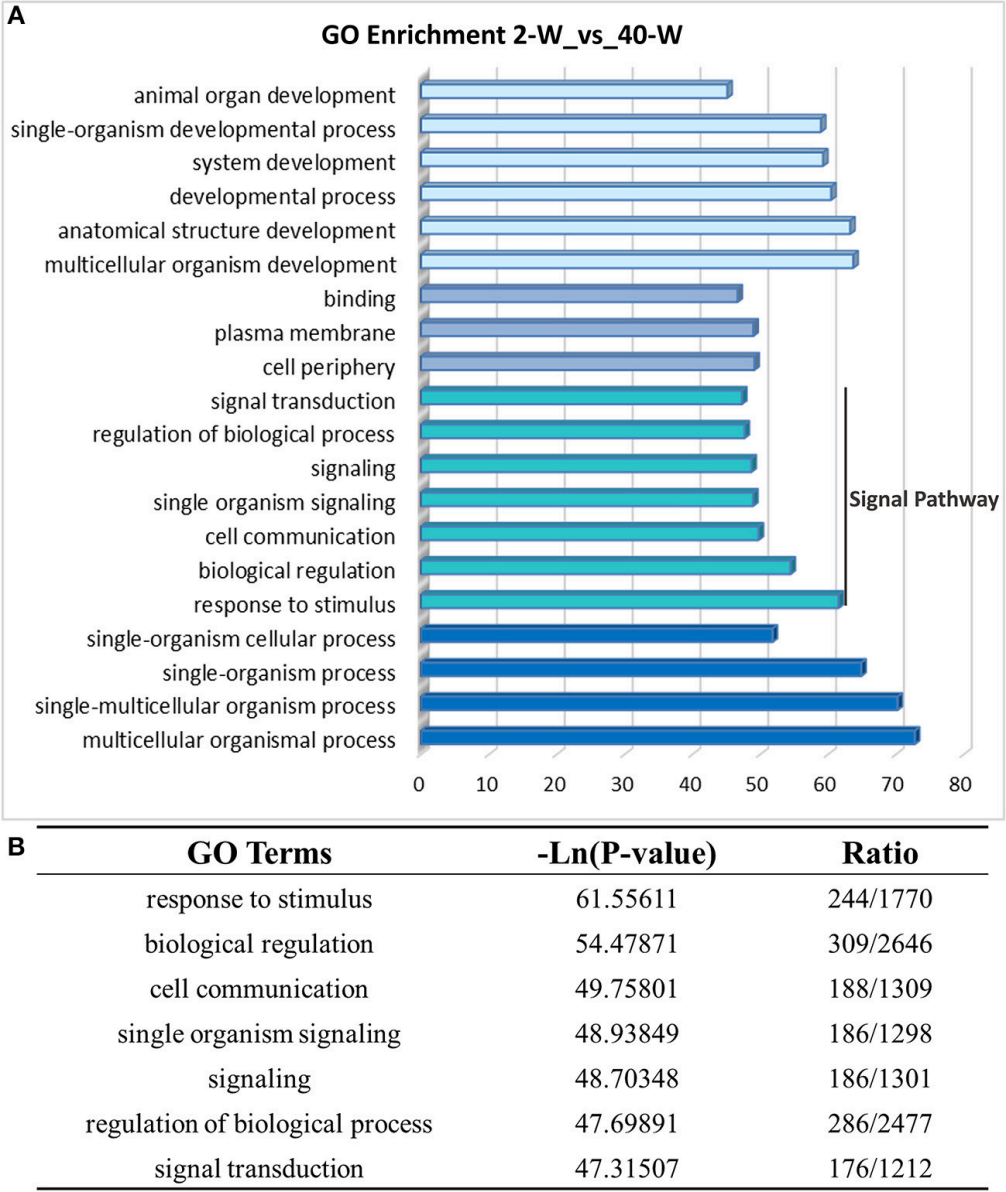
Gene ontology enrichment analysis of DEGs screened from 40-W group and 2-W group. The 20 top gene ontology terms significantly enriched were shown in Figure 3A, and 7 of these 20 terms were related to signal transduction and biological regulation **(B)**.

**Figure 4:**
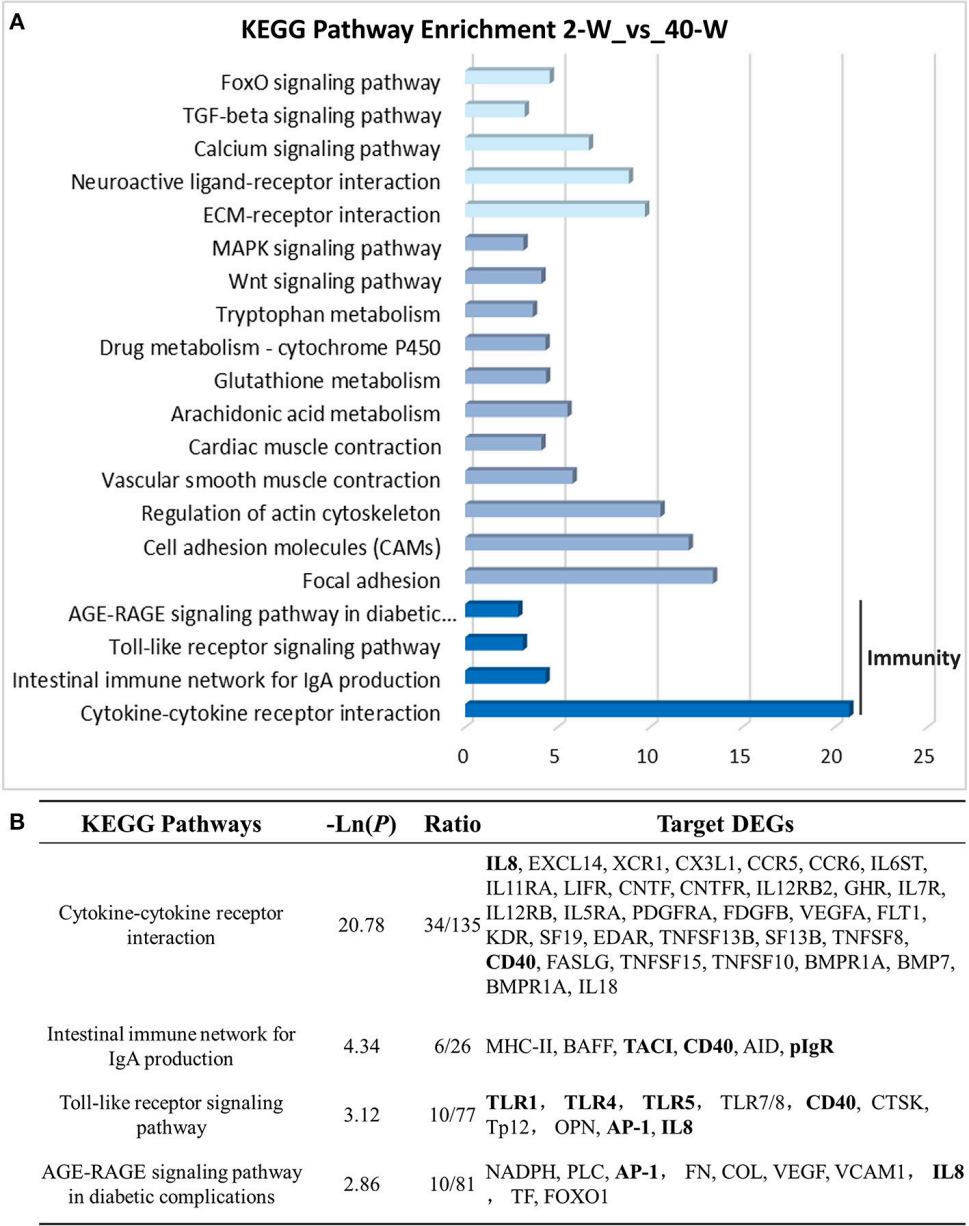
KEGG pathway enrichment analysis of DEGs. The 20 top KEGG pathways significantly enriched were shown in **(A)**. Four (including statistical significance and DEGs of the 4 pathways) of these 20 pathways were associated with immune regulation **(B)**.

On the basis of DEGs, GO, and KEGG enrichment analysis were conducted to investigate the effects of developmental stages on thymus immunity of chickens, and the results of enrichment analysis were shown in Figures 3, **4**, respectively. The 20 top canonical GO terms significantly (*p* < 0.05) enriched were shown in Figure 3A, 7 terms (response to stimulus, biological regulation, cell communication, single organism signaling, signaling, regulation of biological process, signal transduction) of these 20 enriched GO terms were in relation to signal transduction and biological regulation (Figure 3B). To learn the canonical pathways related to thymus developmental stages of chicken, we found out the relevant pathways within DEGs, and the top 20 significantly enriched KEGG pathways were showed in Figure 4A. Among these 20 enriched pathways, 4 pathways (Cytokine-cytokine receptor interaction, Intestinal immune network for IgA production, Toll-like receptor signaling pathway, AGE-RAGE signaling pathway in diabetic complications) were closely associated with immune regulation (Figure 4B). Through GO and KEGG pathway enrichment analysis, we further confirmed that thymus immunity of chickens had a strong age-related correlation.

### Expression data validation using quantitative real time PCR (qRT-PCR)

**Figure 5:**
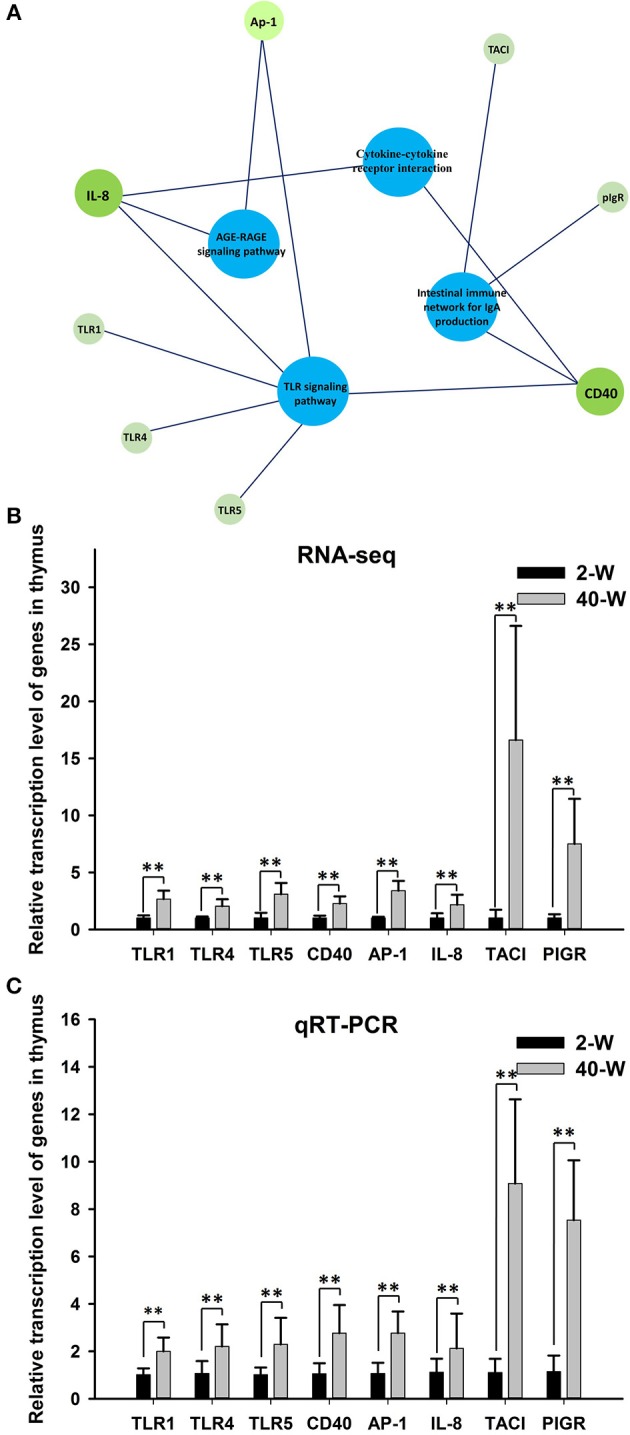
Expression data validation using quantitative real time PCR (qRT-PCR). The 8 analyzed DEGs (*IL-8, CD40, AP-1, TLR1, TLR4, TLR5, TACI*, and *PIGR*) were closely associated to the 4 enriched immune-related pathway **(A)**. It showed that the relative expression levels of the eight DEGs gotten from qRT-PCR **(C)** were in line with that of RNA-Seq **(B)**, and transcription of the 8 genes were all up-regulated in 40-W group compared to the 2-W group. Data shown as mean ± SD, **P < 0.01.

According to the results of GO and KEGG pathway enrichment analysis, eight DEGs related to immunoregulation, including *TLR4, TLR1, TLR5, CD40, AP-1, IL-8, TACI*, and *PIGR* (Figure 5A), were selected and tested using RT-qPCR method to validate the accuracy of RNA-seq data. The results showed that the relative expression levels of the eight DEGs gotten from qRT-PCR (Figure 5C) were largely in line with that of RNA-Seq (Figure 5B). This demonstrated that the transcription data of RNA-Seq was accurate. Meanwhile, compared with 2-W group, transcription of these eight DEGs were all significantly (*p* < 0.01) up-regulated in 40-W group. It meant that the thymus immunity of 40-W grown chickens was up-regulated, in contrast to the 2-W young chicks.

### Negative correlation between *CD40* transcription and promoter methylation

**Figure 6:**
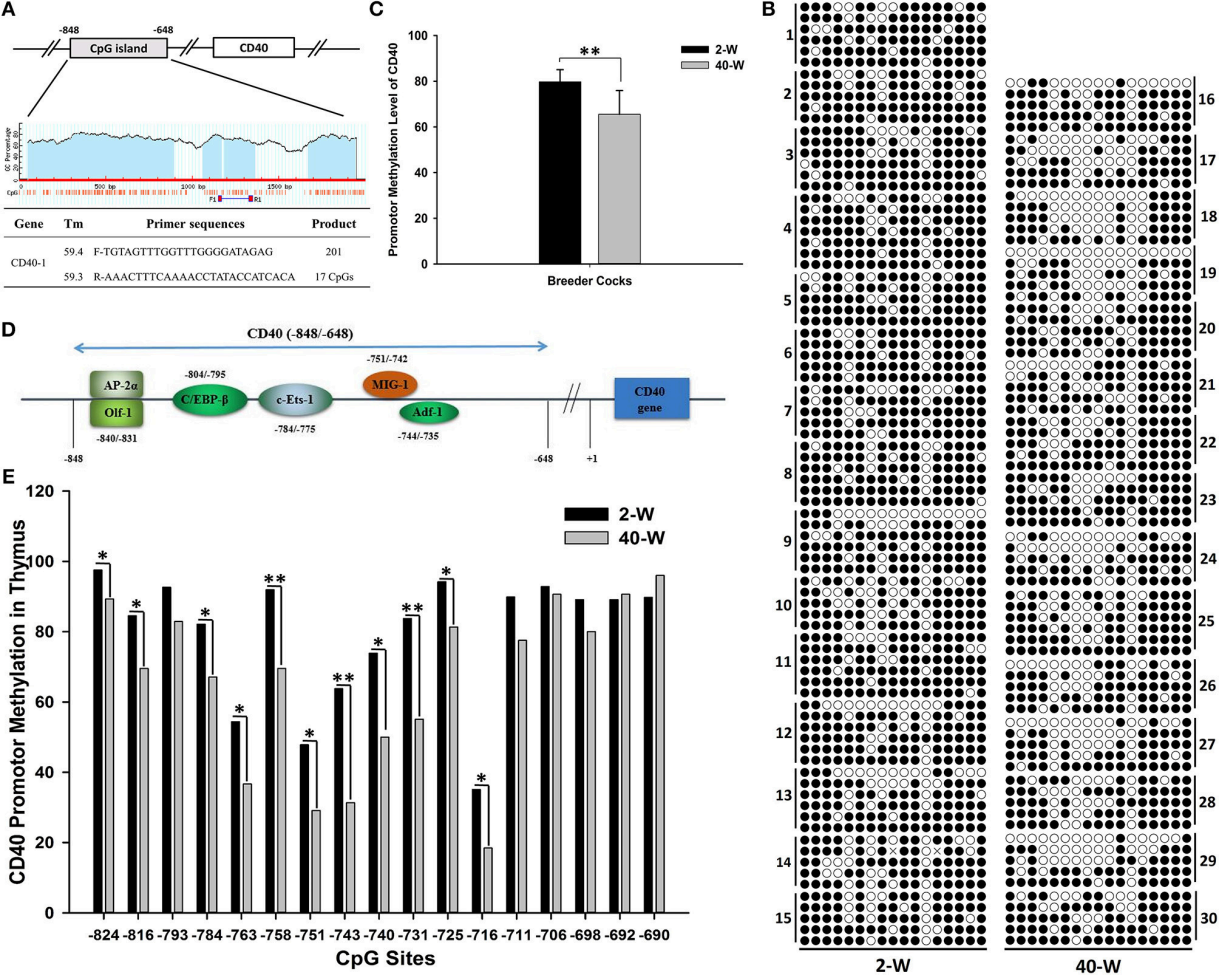
Developmental stages' (2 and 40-weeks-age) effect on promotor methylation of CD40 in thymus. Primer sequences for CD40 promotor methylation were shown as **(A)**, and the promotor CpG island of CD40 contained 201 bases and 17 CpG sites. Results of CD40 promotor methylation were shown in **(B)**. A single raw means a cloned sequence, and a circle means a CpG site. A filled circle means a methylated cytosine, and an open circles means an un-methylated cytosine. *T*-test and histogram of the overall CD40 promotor CpG methylation showed highly significant effects between 2-W and 40-W group **(C)**. Transcription factor binding site prediction showed that the transcription factors with high-affinity binding activity in the region analyzed were all transcription activators **(D)**. Chi-square test and histogram of single CpG site showed that the methylation level of 40-W group was significantly down-regulated in transcription activators-binding domain (−840 ~ −745; **E)**. Data shown as mean ± SD, *P < 0.05, **P < 0.01.

To explore the transcriptional regulatory mechanism responsible for activation of the gene, we analyzed the methylation level of promoter region of *CD40*. The promoter region analyzed was composed of 201 nucleotides, and contained 17 CpGs (Figure 6A). It showed that appropriate level of methylation occurred in *CD40* promoter of thymus, and the promoter methylation level of *CD40* was decreased in 40-W group compared to 2-W group (Figure 6B). *T*-test and histogram of the results indicated that the methylation level was significantly (*p* < 0.01) decreased from 79.77% in 2-W group to 65.52% in 40-W group (Figure 6C). Of all the 17 CpG sites within the promoter region analyzed, compared to the 2-W group, the methylation level of 11 promoter CpG sites were significantly (*p* < 0.05, −824, −816, −784, −763, −751, −740, −725, and −716) or highly significantly (*p* < 0.01, −758, −743, and −731) decreased in the promoter region between −824 and −716 (Figure 6E). Our study suggested there might be a correlation between developmental stages and promoter CpG island methylation of *CD40*.

## Discussion

Thymus plays a pivotal role in the development of the immune system, and it is the primary donor of lymphocyte for the immune system (2). Nevertheless, morphology and immunity of mammals' thymus are varied with age. This is also true for chicken thymus, herein, thymus weight and morphology of chick (2-W) was quite different from that of grown chicken (40-W). According to the thymus' weight and morphology, thymus weight of 2-weeks old chick was much smaller than that of grown chicken, and its thymus morphology was much rounder than the grown chicken's. We can conclude that thymus of the chick undergoes rapid growth. Whereas, as to grown chicken, its thymus index was highly significantly smaller than young chick's, and its thymus morphology was thinner, and much more oval and elongate. And so, thymus of grown chicken has already been in a state of degeneration. Considering these differences, immune-function of thymus would also inevitably be affected. Indeed, researches before indicated that compared to the adult animals, the developing immune system of young animals was characterized by suppressed immune system, blunted inflammatory cytokine production and skewed T and B cell development in favor of regulatory responses (21). However, the detailed effects of developmental stages on chicken thymus immunity are remains to be fully elucidated. And so, transcriptome analysis of thymus was conducted to get a better handle on this problem.

The results of PCC and PCA led us to believe that the whole transcriptional pattern of 2-W group was distinct from that of 40-W group. Considering thymus is an immune organ, these results also proved that thymuses of young chick and grown chicken were of vary different immunity. The detailed biological differences between chick and grown chicken arise from the DEGs. In comparison to the 2-W group, we got 1949 DEGs (1722 up-regulated and 127 down-regulated) from the 40-W group. To understand the possible immune functions associated with these DEGs observed in the 40-W group vs. the 2-W group, GO, and KEGG enrichment analysis was adopted. We found 4 significantly enriched KEGG signaling pathways which were crucial for immune regulation. These screened pathways, including cytokine-cytokine receptor interaction, intestinal immune network for IgA production, Toll-like receptor signaling pathway, AGE-RAGE signaling pathway in diabetic complications, could partly explain the correspondence between developmental stages and thymus immunity. Toll-like receptors are the main receptor proteins located on the surface of immune cells, and tasked with recognizing conserved structures of intruders (22). This suggests, Toll-like receptor signaling pathway is crucial for modulating innate and adaptive immune responses to infected pathogens, as well as endogenous antigens (23–25). While, cytokine-cytokine receptor interaction is involved in recognition of cytokine and chemotactic factor, and further controlling the hematopoietic cells' differentiation, proliferation, migration, or to perform bio-functions such as phagocytosis or activate inflammatory response (26, 27). Another significantly enriched pathway, AGE-RAGE signaling pathway in diabetic complications, as well, has a close relation with inflammatory response (28). Last but not least, intestinal immune network for IgA production, is mainly implicated in the intestinal adaptive immune response to gut microbiota, and specifically reducing the growth of some microbes (29–31). Transcription of 8 screened DEGs (as well as core regulators of these significantly enriched immune pathways) in 40-W group were up-regulated compared with that of 2-W group. Other DEGs in these 4 signaling pathway exhibited consistency change between 2-W and 40-W group. This means that, in contrast to 2-W group, DEGs enriched in these 4 screened KEGG pathways were all hyper-activated in 40-W group. It indicated that thymus immunity of 2-weeks-age chick, mainly about immune recognition and signaling transduction, intestinal adaptive immune response to gut microbiota, and regulation of inflammatory response, was kept in a status of hypo-activation. A possible explanation to these results is that thymus immunity is varied with age, and this variance is related to the co-adaptation between chicken's thymus immunity and intestinal microbiota.

Age is one of the major drivers of changes in gut microbiota (32, 33). For chicken, its intestine contained significantly different microbiome composition through the life span, but the intestinal microbiome showed clear successional changes as the bird matured (34–36). The host immune system is frequently described as being “tolerant” to the high numbers of bacteria that symbiotic with the host (32, 37). While, for young animals, the neonate innate immune system can't successfully deal with trillions of intestinal microbiotas. To survive from various intestinal pathogens, young animals must have more “patient” than adult animals. For example, in the intestine of young animals, more mucosal mucin must be secreted to shield host from intestinal microbiota (37, 38). Even if, some intestinal microorganisms can be directly in contact with intestinal epithelial cells and recognized by pattern-recognition receptors (PRR, such as TLRs), the induced immune response to these microorganisms is highly distinct from that of adult animals, with notable impairment in the production of inflammatory mediators such as pro-inflammatory cytokines and oxygen radicals, but heightened production of regulatory cytokines such as IL-10 (32, 39). For young animals, the primary fraction of the immune system's constitutive function is to control the interaction between animals and intestinal microbiota, and have the characteristics of a reduced number of T cells and intestinal IgA-production (32). In our research, as well, thymus immunity, including antigen recognition, intestinal IgA-production, and inflammation of 2-weeks-age chick, was down-regulated compared to those of 40-weeks-age chicken.

Hypo-activation of chick thymus immunity in young age is modulated by the corresponding DEGs, and this transcriptional pattern could sustain a rather long time (at least 2 weeks). Among all kinds of regulatory mechanisms of gene expression, only epigenetics modification can meet this requirement. Epigenetic modifications, including DNA methylation, histone covalent modifications, chromosome reconstruction, and micro-RNA, have a common characteristic, high in stability, and the status of epigenetic modification can be maintained for a long time. DNA methylation is a kind of gradient mechanism in regulating gene expression by a time-dependent manner, and it is an important factor that responsible for establishment, maintenance, and reversal of metastable transcriptional states (40). Many researches before found that DNA methylation is also critical in the coadaptation between symbiotic microorganisms and the host (41–45), and modulation of immune cell differentiation (46–48). It means that there exist microbiota-dependent epigenetic programming of genes related to intestinal mucosal immunity and systematic immunity, which is critical for animal health and intestinal function. So we hypothesized that the thymus immunity varied with age was modulated by promotor methylation of core regulators.

CD40 is one of the 8 core regulators, and it closely related to 3 (cytokine-cytokine receptor interaction, intestinal immune network for IgA production, and Toll-like receptor signaling pathway) of the 4 screened pathways. In addition, compared with the other 7 DEGs, the promoter CpG island of CD40 contains a maximum amount of CpG sites (17 CpG sites in the region analyzed). CD40 is an important transmembrane receptor which is widely expressed in almost all kinds of immune cells, including T/B cells, dendritic cells, monocytes, and macrophages, and it plays a critical role in immunity and inflammation (49–51). In the process of host immune-regulation, CD40 pathway is involved in both cellular immunity and humoral immunity. By interaction with CD40L, CD40 could modulate the crosstalk between antigen presenting cells (macrophages and dendritic cell) and T/B cell, and further induce the expression of pro-inflammatory cytokines and activation of B cell response (52), as well as synthesis of IgG/A (50, 53). As a key co-stimulatory molecule in T cell, CD40 is critical for body's defense against invading pathogens, and plays essential role in adaptive immunity by eliciting survival, proliferation, differentiation and/or cell death signaling pathways (54, 55), and so, contributes much to immune response against pathogen infection (56), however, these immune responses often accompany inflammatory response (56–58). CD40 and CD40 ligand (CD40L) participate in numerous inflammatory pathways, as well as oxidative stress (50). Studies in monocytes and macrophages revealed that activation of CD40 would result in a primarily pro-inflammatory response, and efficiently activate the synthesis of pro-inflammatory cytokines (e.g., IL-1, IL-8, TNF-α, IL-12, IL-6, CCL2, and so on) (59). Above all, CD40 is critical for host immunity, and down-regulation of *CD40* had a certain correlation with suppression of thymus immunity.

So we analyzed the promoter methylation of *CD40* by using BSP method to learn if it existed differences in *CD40* promoter methylation between 2-W group and 40-W group. The results showed that the *CD40* promoter methylation was significant different between 2-W group and 40-W group, and it was significantly down-regulated in the 40-W group. Previous studies on DNA methylation have found that methylated CpG dinucleotide can be recognized and bound by the proteins with a methyl-CpG-binding domain (MBD), such as methyl-CpG-binding protein 2, MBD1, MBD2, and MBD4 (16, 60, 61). These MBD-containing proteins can compete with transcription factors for non-sequence-specific DNA binding, and controlling the genes' expression at the transcriptional level (15). Results of transcription factor binding site prediction showed that the transcription factors with high-affinity binding activity in *CD40* gene promoter region (−840 ~ −735) were all transcription activators (Figure 6D). Methylation level of 11 CpG sites in the region between −824 and −716 was significantly increased in 2-W group. And so, hyper-methylation of *CD40* promotor in 2-W group can inhibit the recruit of transcription activators, and stabilize and suppress the transcription of *CD40*. Given that DNA (de)methylation modification is a time-consuming biochemical reaction. Hyper-methylation of *CD40* promotor in chick thymus can stabilize the low expression of *CD40*, and further contribute to the hypo-activation of thymus immunity.

From the discussion above, we can draw the conclusion that thymus immunity of chicken is varied with age. Thymus immunity of chick, including antigen recognition and presentation, intestinal IgA-production, and inflammation, was inhibited in comparison to that of grown chicken. Suppression of *CD40*, which is induced by promotor hyper-methylation, might be critical for the maintenance of hypo-activated immune status in chick thymus. But more research should be conducted to further analyze the internal relation between gut microbiota and thymus immunity, and the regulatory effects of CD40 in chicken's immune response to gut microbiota.

## Data availability

The authors confirm that all data underlying the findings are fully available without restriction.

## Author contributions

YL, XL, ZY, QS, and XY conceived and designed the experiments. YL, XL, WG, and SW performed the experiments. YL and XL analyzed the data. XY contributed reagents, materials, analysis tools. YL wrote the manuscript. XY and HL provided editorial suggestions and revisions.

### Conflict of interest statement

The authors declare that the research was conducted in the absence of any commercial or financial relationships that could be construed as a potential conflict of interest.

## Supplementary material

Table S1 Ingredient and nutrient composition of breeder cocks' basal diet. ^1^Apparent Metabolizable Energy; ^2^Kilocalorie.

Table S2 Primer sequences for quantitative real-time PCR analysis.

Table S3 Quality of the total RNA. ^1^RNA integrity number.

Table S4 Overview of the RNA-Seq library.

Table S5 RPKMS of all the unigenes.

Figure S1 Agrose gel electrophoresis of BSP product. The BSP product showed a clear band in 2% agarose gels, and it contained about 200 bases.

Figure S2 The base percentage composition along raw reads.

Figure S3 The quality distribution of bases along reads.

Figure S4 Randomness assessment of the samples.

## References

[1] von Boehmer H. The thymus in immunity and in malignancy. Cancer Immunol Res. (2014) 2:592–7. 10.1158/2326-6066.CIR-14-0070 24990239

[2] Zdrojewicz Z Pachura E Pachura P. The thymus: a forgotten, but very important organ. Adv Clin Exp Med. (2016) 25:369–75. 10.17219/acem/58802 27627572

[3] Bunting MD Comerford I McColl SR. Finding their niche: chemokines directing cell migration in the thymus. Immunol Cell Biol. (2011) 89:185–96. 10.1038/icb.2010.142 21135866

[4] Ma D Wei Y Liu F. Regulatory mechanisms of thymus and T cell development. Dev Comp Immunol. (2013) 39:91–102. 10.1016/j.dci.2011.12.013 22227346

[5] Rezzani R Nardo L Favero G Peroni M Rodella LF. Thymus and aging: morphological, radiological, and functional overview. Age (2014) 36:313–51. 10.1007/s11357-013-9564-5 23877171 PMC3889907

[6] Stritesky GL Jameson SC Hogquist KA. Selection of self-reactive T cells in the thymus. Annu Rev Immunol. (2012) 30:95–114. 10.1146/annurev-immunol-020711-075035 22149933 PMC3518413

[7] Carpenter AC Bosselut R. Decision checkpoints in the thymus. Nat Immunol. (2010) 11:666–73. 10.1038/ni.1887 20644572 PMC3388799

[8] Kondo K Takada K Takahama Y. Antigen processing and presentation in the thymus: implications for T cell repertoire selection. Curr Opin Immunol. (2017) 46:53–7. 10.1016/j.coi.2017.03.014 28477557

[9] Klein L Hinterberger M Wirnsberger G Kyewski B. Antigen presentation in the thymus for positive selection and central tolerance induction. Nat Rev Immunol. (2009) 9:833–44. 10.1038/nri2669 19935803

[10] Aspinall R Pitts D Lapenna A Mitchell W. Immunity in the elderly: the role of the thymus. J Comp Pathol. (2010) 142(Suppl 1):S111–5. 10.1016/j.jcpa.2009.10.022 19954794

[11] Goronzy JJ Weyand CM. Successful and maladaptive T cell aging. Immunity (2017) 46:364–78. 10.1016/j.immuni.2017.03.010 28329703 PMC5433436

[12] Kumar BV Connors TJ Farber DL. Human T cell development, localization, and function throughout life. Immunity (2018) 48:202–13. 10.1016/j.immuni.2018.01.007 29466753 PMC5826622

[13] Moore LD Le T Fan G. DNA methylation and its basic function. Neuropsychopharmacol (2012) 38:23–38. 10.1038/npp.2012.112 22781841 PMC3521964

[14] Zhang H Lang Z Zhu JK. Dynamics and function of DNA methylation in plants. Nat Rev Mol Cell Biol. (2018) 19:489–506. 10.1038/s41580-018-0016-z 29784956

[15] Jones PA. Functions of DNA methylation: islands, start sites, gene bodies and beyond. Nat Rev Genet. (2012) 13:484–92. 10.1038/nrg3230 22641018

[16] Wu H Zhang Y. Reversing DNA methylation: mechanisms, genomics, and biological functions. Cell (2014) 156:45–68. 10.1016/j.cell.2013.12.019 24439369 PMC3938284

[17] Schübeler D. Function and information content of DNA methylation. Nature (2015) 517:321–6. 10.1038/nature14192 25592537

[18] Kanehisa M Araki M Goto S Hattori M Hirakawa M Itoh M. KEGG for linking genomes to life and the environment. Nucleic Acids Res. (2008) 36:D480–4. 10.1093/nar/gkm882 18077471 PMC2238879

[19] Li L-C Dahiya R. MethPrimer: designing primers for methylation PCRs. Bioinformatics (2002) 18:1427–31. 10.1093/bioinformatics/18.11.1427 12424112

[20] Kumaki Y Oda M Okano M. QUMA: quantification tool for methylation analysis. Nucleic Acids Res. (2008) 36:W170–5. 10.1093/nar/gkn294 18487274 PMC2447804

[21] PrabhuDas M Adkins B Gans H King C Levy O Ramilo O. Challenges in infant immunity: implications for responses to infection and vaccines. Nat Immunol. (2011) 12:189–94. 10.1038/ni0311-189 21321588

[22] Lim KH Staudt LM. Toll-like receptor signaling. Cold Spring Harb Perspect Biol. (2013) 5:a011247. 10.1101/cshperspect.a011247 23284045 PMC3579400

[23] Akira S Takeda K Toll-like receptor signalling. Nat Rev Immunol. (2004) 4:499 10.1038/nri1391 15229469

[24] Iwasaki A Medzhitov R. Toll-like receptor control of the adaptive immune responses. Nat Immunol. (2004) 5:987. 10.1038/ni1112 15454922

[25] Kawai T Akira S. The role of pattern-recognition receptors in innate immunity: update on Toll-like receptors. Nat Immunol. (2010) 11:373. 10.1038/ni.1863 20404851

[26] Lopez AF Hercus TR Ekert P Littler DR Guthridge M Thomas D. Molecular basis of cytokine receptor activation. IUBMB Life (2010) 62:509–18. 10.1002/iub.350 20540154

[27] Broughton SE Hercus TR Lopez AF Parker MW. Cytokine receptor activation at the cell surface. Curr Opin Struc Biol. (2012) 22:350–9. 10.1016/j.sbi.2012.03.015 22521507

[28] Ramasamy R Yan SF Schmidt AM. Receptor for AGE (RAGE): signaling mechanisms in the pathogenesis of diabetes and its complications. Ann N Y Acad Sci. (2011) 1243:88–102. 10.1111/j.1749-6632.2011.06320.x 22211895 PMC4501013

[29] Macpherson AJ McCoy KD Johansen FE Brandtzaeg P. The immune geography of IgA induction and function. Mucosal Immunol. (2007) 1:11–22. 10.1038/mi.2007.6 19079156

[30] Maynard CL Elson CO Hatton RD Weaver CT. Reciprocal interactions of the intestinal microbiota and immune system. Nature (2012) 489:231–41. 10.1038/nature11551 22972296 PMC4492337

[31] McDermott AJ Huffnagle GB. The microbiome and regulation of mucosal immunity. Immunology (2014) 142:24–31. 10.1111/imm.12231 24329495 PMC3992044

[32] Belkaid Y Hand TW. Role of the microbiota in immunity and inflammation. Cell (2014) 157:121–41. 10.1016/j.cell.2014.03.011 24679531 PMC4056765

[33] Belkaid Y Harrison OJ. Homeostatic immunity and the microbiota. Immunity (2017) 46:562–76. 10.1016/j.immuni.2017.04.008 28423337 PMC5604871

[34] Lu J Idris U Harmon B Hofacre C Maurer JJ Lee MD. Diversity and succession of the intestinal bacterial community of the maturing broiler chicken. Appl Environ Microb. (2003) 69:6816–24. 10.1128/AEM.69.11.6816-6824.2003 14602645 PMC262306

[35] Yeoman CJ Chia N Jeraldo P Sipos M Goldenfeld ND White BA. The microbiome of the chicken gastrointestinal tract. Anim Health Res Rev. (2012) 13:89–99. 10.1017/S1466252312000138 22853945

[36] Oakley BB Lillehoj HS Kogut MH Kim WK Maurer JJ Pedroso A. The chicken gastrointestinal microbiome. FEMS Microbiol Lett. (2014) 360:100–12. 10.1111/1574-6968.12608 25263745

[37] Duerkop BA Vaishnava S Hooper LV. Immune responses to the microbiota at the intestinal mucosal surface. Immunity (2009) 31:368–76. 10.1016/j.immuni.2009.08.009 19766080

[38] Okumura R Takeda K. Roles of intestinal epithelial cells in the maintenance of gut homeostasis. Exp Mol Med. (2017) 49:e338. 10.1038/emm.2017.20 28546564 PMC5454438

[39] Kollmann TR Levy O Montgomery RR Goriely S. Innate immune function by Toll-like receptors: distinct responses in newborns and the elderly. Immunity (2012) 37:771–83. 10.1016/j.immuni.2012.10.014 23159225 PMC3538030

[40] Bonasio R Tu S Reinberg D. Molecular signals of epigenetic states. Science (2010) 330:612–6. 10.1126/science.1191078 21030644 PMC3772643

[41] Raghuraman S Donkin I Versteyhe S Barres R Simar D. The emerging role of epigenetics in inflammation and immunometabolism. Trends Endocrinol Metab. (2016) 27:782–95. 10.1016/j.tem.2016.06.008 27444065

[42] Robert McMaster W Morrison CJ Kobor MS. Epigenetics: a new model for intracellular parasite-host cell regulation. Trends Parasitol. (2016) 32:515–21. 10.1016/j.pt.2016.04.002 27142564

[43] Thaiss CA Zmora N Levy M Elinav E. The microbiome and innate immunity. Nature (2016) 535:65–74. 10.1038/nature18847 27383981

[44] Blacher E Levy M Tatirovsky E Elinav E. Microbiome-modulated metabolites at the interface of host immunity. J Immunol. (2017) 198:572–80. 10.4049/jimmunol.1601247 28069752

[45] Pan X Gong D Nguyen DN Zhang X Hu Q Lu H Early microbial colonization affects DNA methylation of genes related to intestinal immunity and metabolism in preterm pigs. DNA Res. (2018) 25:287–96. 10.1093/dnares/dsy001 PMC6014285 29365082

[46] Chen L Ge B Casale FP Vasquez L Kwan T Garrido-Martin D. Genetic drivers of epigenetic and transcriptional variation in human immune cells. Cell (2016) 167:1398–414 e1324. 10.1016/j.cell.2016.10.026 27863251 PMC5119954

[47] Wang H Wang J Ning C Zheng X Fu J Wang A. Genome-wide DNA methylation and transcriptome analyses reveal genes involved in immune responses of pig peripheral blood mononuclear cells to poly I:C. Sci Rep. (2017) 7:9709. 10.1038/s41598-017-10648-9 28852164 PMC5575306

[48] Barwick BG Scharer CD Martinez RJ Price MJ Wein AN Haines RR. B cell activation and plasma cell differentiation are inhibited by *de novo* DNA methylation. Nat Commun. (2018) 9:1900. 10.1038/s41467-018-04234-4 29765016 PMC5953949

[49] Tong AW Stone MJ. Prospects for CD40-directed experimental therapy of human cancer. Cancer Gene Ther. (2002) 10:1–13. 10.1038/sj.cgt.7700527 12489023

[50] Rizvi M Pathak D Freedman JE Chakrabarti S. CD40-CD40 ligand interactions in oxidative stress, inflammation and vascular disease. Trends Mol Med. (2008) 14:530–8. 10.1016/j.molmed.2008.09.006 18977174

[51] Byrne KT Vonderheide RH. CD40 Stimulation obviates innate sensors and drives T cell immunity in cancer. Cell Rep. (2016) 15:2719–32. 10.1016/j.celrep.2016.05.058 27292635 PMC4917417

[52] Jain S Chodisetti SB Agrewala JN. CD40 Signaling Synergizes with TLR-2 in the BCR Independent Activation of Resting B Cells. PLOS ONE (2011) 6:e20651. 10.1371/journal.pone.0020651 21674065 PMC3107243

[53] Ma DY Clark EA. The role of CD40 and CD154/CD40L in dendritic cells. Semin Immunol (2009) 21:265–72. 10.1016/j.smim.2009.05.010. 19524453 PMC2749083

[54] Elgueta R Benson MJ De Vries VC Wasiuk A Guo Y Noelle RJ. Molecular mechanism and function of CD40/CD40L engagement in the immune system. Immunol Rev. (2009) 229:152–72. 10.1111/j.1600-065X.2009.00782.x 19426221 PMC3826168

[55] Smulski CR Decossas M Chekkat N Beyrath J Willen L Guichard G. Hetero-oligomerization between the TNF receptor superfamily members CD40, Fas and TRAILR2 modulate CD40 signalling. Cell Death Dis. (2017) 8:e2601. 10.1038/cddis.2017.22 28182009 PMC5386471

[56] Munroe ME. Functional roles for T cell CD40 in infection and autoimmune disease: the role of CD40 in lymphocyte homeostasis. Semin Immunol. (2009) 21:283–8. 10.1016/j.smim.2009.05.008 19539498

[57] Pietrella D Lupo P Perito S Mosci P Bistoni F Vecchiarelli A. Disruption of CD40/CD40L interaction influences the course of *Cryptococcus neoformans* infection. FEMS Immunol Med Microbiol. (2004) 40:63–70. 10.1016/S0928-8244(03)00297-9 14734188

[58] Subauste CS. CD40 and the immune response to parasitic infections. Semin Immunol. (2009) 21:273–82. 10.1016/j.smim.2009.06.003 19616968 PMC2758065

[59] Suttles J Stout RD. Macrophage CD40 signaling: a pivotal regulator of disease protection and pathogenesis. Semin Immunol. (2009) 21:257–64. 10.1016/j.smim.2009.05.011 19540774

[60] Zou X Ma W Solov'yov IA Chipot C Schulten K. Recognition of methylated DNA through methyl-CpG binding domain proteins. Nucleic Acids Res. (2012) 40:2747–58. 10.1093/nar/gkr1057 22110028 PMC3315304

[61] Zhu H Wang G Qian J. Transcription factors as readers and effectors of DNA methylation. Nat Rev Genet. (2016) 17:551–65. 10.1038/nrg.2016.83 27479905 PMC5559737

