# Supplementary material for: Age-Related Changes on CD40 Promotor Methylation and Immune Gene Expressions in Thymus of Chicken
Source: Front Immunol. 2018 Nov 21;9:2731. doi: 10.3389/fimmu.2018.02731 (PMC6259354; doi:10.3389/fimmu.2018.02731)
Supplement: Table S1 — Ingredient and nutrient composition of breeder cocks' basal diet. 1Apparent Metabolizable Energy; 2Kilocalorie. [file Table_1.docx]

**SUPPLEMENTARY TABLE 1**. **Ingredient and nutrient composition of chicken’s basal diet.** ^1^ Apparent Metabolizable Energy; ^2^ Kilocalorie

| Item | Composition | |
| --- | --- | --- |
|  | 1-4 w | 5-40 w |
| Maize, % | 64 | 63 |
| Soybean meal (43% protein), % | 28 | 25 |
| Wheat bran, % | 2.41 | 6.6 |
| Limestone, % | 1.6 | 1.5 |
| Dicalcium phosphate, % | 1.4 | 1.4 |
| Soybean oil, % | 1 | 1 |
| Vitamin and mineral mix, % | 0.99 | 0.99 |
| Salt, % | 0.28 | 0.3 |
| Methionine, % | 0.132 | 0.07 |
| Choline chloride, % | 0.1 | 0.08 |
| Lysine, % | 0.05 | 0.05 |
| Phytase, % | 0.01 | 0.01 |
| Calculated nutrient composition |  |  |
| AME^1^, KC^2^ | 2900 | 2864 |
| Crude protein, % | 19 | 18 |
| Calcium, % | 0.97 | 0.93 |
| Non-phytate phosphorus, % | 0.38 | 0.36 |
| Total phosphorus, % | 0.56 | 0.58 |
| Salt, % | 0.31 | 0.34 |
| Lysine, % | 1.01 | 0.896 |
| Methionine, % | 0.434 | 0.365 |
| Met+Cys, % | 0.76 | 0.676 |
| Isoleucine, % | 0.713 | 0.664 |
| Threonine, % | 0.726 | 0.682 |
| Tryptophan, % | 0.234 | 0.215 |
| Valnine, % | 0.857 | 0.806 |
| Argnine, % | 1.207 | 1.113 |
