# Supplementary material for: Age-Related Changes on CD40 Promotor Methylation and Immune Gene Expressions in Thymus of Chicken
Source: Front Immunol. 2018 Nov 21;9:2731. doi: 10.3389/fimmu.2018.02731 (PMC6259354; doi:10.3389/fimmu.2018.02731)
Supplement: Table S2 — Primer sequences for quantitative real-time PCR analysis. [file Table_2.docx]

**SUPPLEMENTARY TABLE 2.** **Primer sequences for quantitative real-time PCR analysis.**

| Gene | ID of mRNA | Primer Sequence（5'-3'） | Product/ bp |
| --- | --- | --- | --- |
| TLR1 | NM_001007488.4 | F-CTGGGCAAAACCCGTTCAAG | 161 |
|  |  | R-CAGCTCGGTCAAGTGGAAGT |  |
| TLR4 | NM_001030693.1 | F-GGCTCAACCTCACGTTGGTA | 220 |
|  |  | R-AGTCCGTTCTGAAATGCCGT |  |
| TLR5 | NM_001024586.1 | F-CACCTGCATTAGCGGGTGTA | 87 |
|  |  | R-CCGAGAACCTGAGTGTCTGC |  |
| CD40 | NM_204665.2 | F-TGCACACCCTGTGAGAATGG | 129 |
|  |  | R-AGTGTTGTGCGTTGCGTTTC |  |
| AP-1 | NM_001031289.1 | F-CGAAAGCGCATGTGTGGAAA | 105 |
|  |  | R-GATCTGACGCCTCATCGGAG |  |
| IL-8 | NM_205498.1 | F-TGGTCAGTGCTGTGGGATTC | 76 |
|  |  | R-TCATTTCCCCTAGCAAGCCC |  |
| TACI | NM_001097537.1 | F-CATCCCAGTTCCTCCATCGG | 126 |
|  |  | R-AGCTCCTCTGTTCTTGTGCG |  |
| PIGR | NM_001044644.1 | F-GGATTATGCCAGCACGAGGA | 153 |
|  |  | R-GTGATCATGACGCTGAACGC |  |
| β-actin | L08165 | F-ATTGTCCACGCAAATGCTTC | 113 |
|  |  | R-AAATAAAGCCATGCCAACTCGTC |  |
