# Supplementary material for: Age-Related Changes on CD40 Promotor Methylation and Immune Gene Expressions in Thymus of Chicken
Source: Front Immunol. 2018 Nov 21;9:2731. doi: 10.3389/fimmu.2018.02731 (PMC6259354; doi:10.3389/fimmu.2018.02731)
Supplement: Table S3 — Quality of the total RNA. 1RNA integrity number. [file Table_3.docx]

**SUPPLEMENTARY TABLE 3. Quality of the total RNA.** ^1^ RNA integrity number

| Samples | RNA concentration (ng/uL) | | RNA Amount (ug) | RIN^1^ | | 28S/18S |
| --- | --- | --- | --- | --- | --- | --- |
| 1 | | 1440 | 288 | 9.3 | 1.4 | |
| 2 | | 1602 | 320.4 | 9.5 | 1.4 | |
| 3 | | 1440 | 288 | 9.3 | 1.4 | |
| 4 | | 1728 | 345.6 | 8.3 | 1 | |
| 5 | | 1935 | 387 | 8.9 | 1.3 | |
| 6 | | 1477 | 295.4 | 9.3 | 1.4 | |
| 7 | | 1312 | 262.4 | 9.3 | 1.3 | |
| 8 | | 1200 | 240 | 8.8 | 1.2 | |
| 9 | | 1211 | 242.2 | 9 | 1.2 | |
| 10 | | 1376 | 275.2 | 8.6 | 1.1 | |
| 11 | | 2070 | 414 | 9 | 1.2 | |
| 12 | | 1890 | 378 | 9.3 | 1.3 | |
| 13 | | 2259 | 451.8 | 8.8 | 1.2 | |
| 14 | | 1840 | 368 | 8.6 | 1 | |
| 15 | | 2490 | 498 | 8.6 | 1.1 | |
| 16 | | 1920 | 556.8 | 8.7 | 1.3 | |
| 17 | | 1832 | 421.36 | 8.9 | 1.3 | |
| 18 | | 2420 | 338.8 | 8.4 | 1.3 | |
| 19 | | 2700 | 607.5 | 8.8 | 1.3 | |
| 20 | | 2240 | 548.8 | 9 | 1.3 | |
| 21 | | 1862 | 428.26 | 9.2 | 1.4 | |
| 22 | | 1325 | 304.75 | 8.6 | 1.4 | |
| 23 | | 2163 | 551.57 | 9.1 | 1.5 | |
| 24 | | 3390 | 779.7 | 9.3 | 1.5 | |
| 25 | | 1368 | 465.12 | 8.5 | 1.4 | |
| 26 | | 1312 | 314.88 | 8.9 | 1.3 | |
| 27 | | 1376 | 206.4 | 9 | 1.3 | |
| 28 | | 1881 | 263.34 | 9.2 | 1.3 | |
| 29 | | 1413 | 324.99 | 9.2 | 1.4 | |
| 30 | | 1746 | 226.98 | 8.8 | 1.3 | |
