# Supplementary material for: Age-Related Changes on CD40 Promotor Methylation and Immune Gene Expressions in Thymus of Chicken
Source: Front Immunol. 2018 Nov 21;9:2731. doi: 10.3389/fimmu.2018.02731 (PMC6259354; doi:10.3389/fimmu.2018.02731)
Supplement: Table S4 — Overview of the RNA-Seq library. [file Table_4.docx]

**SUPPLEMENTARY TABLE 4**. **Overview of the RNA-Seq library.**

| Sample Name | Clean reads | Genome map Rate | | Gene map Rate | Expressed Gene |
| --- | --- | --- | --- | --- | --- |
| 1 | 46640776 | | 67.30% | 53.71% | 22450 |
| 2 | 46851054 | | 70.91% | 53.70% | 22107 |
| 3 | 46944724 | | 71.06% | 53.53% | 22621 |
| 4 | 48750560 | | 69.29% | 50.25% | 22710 |
| 5 | 46815482 | | 70.05% | 50.82% | 22814 |
| 6 | 46980472 | | 71.67% | 53.09% | 22495 |
| 7 | 48944590 | | 71.71% | 53.49% | 22852 |
| 8 | 46759096 | | 70.79% | 50.97% | 22417 |
| 9 | 49031456 | | 72.05% | 54.31% | 22433 |
| 10 | 46888528 | | 70.61% | 52.91% | 22169 |
| 11 | 46969114 | | 71.65% | 53.87% | 22851 |
| 12 | 48752602 | | 71.06% | 53.82% | 22360 |
| 13 | 46784032 | | 69.97% | 51.84% | 22275 |
| 14 | 46800894 | | 70.29% | 51.44% | 22740 |
| 15 | 48998446 | | 72.83% | 55.43% | 22341 |
| 16 | 48350594 | | 70.26% | 50.89% | 22831 |
| 17 | 48754620 | | 72.34% | 53.93% | 24294 |
| 18 | 48804710 | | 69.94% | 51.64% | 23323 |
| 19 | 48450996 | | 70.76% | 51.38% | 23623 |
| 20 | 48791916 | | 71.93% | 53.62% | 24169 |
| 21 | 48789188 | | 69.31% | 50.09% | 23392 |
| 22 | 48500216 | | 71.68% | 53.15% | 23620 |
| 23 | 48111906 | | 69.44% | 51.16% | 23516 |
| 24 | 48667670 | | 69.73% | 51.11% | 22950 |
| 25 | 48593764 | | 70.21% | 52.71% | 23916 |
| 26 | 48217298 | | 70.95% | 52.72% | 23230 |
| 27 | 49032938 | | 71.09% | 52.51% | 23559 |
| 28 | 48598234 | | 71.69% | 52.42% | 23925 |
| 29 | 48812196 | | 70.72% | 52.83% | 23217 |
| 30 | 48970660 | | 71.06% | 50.97% | 22958 |
