# Supplementary figures and images for: Age-Related Changes on CD40 Promotor Methylation and Immune Gene Expressions in Thymus of Chicken
Source: Front Immunol. 2018 Nov 21;9:2731. doi: 10.3389/fimmu.2018.02731 (PMC6259354; doi:10.3389/fimmu.2018.02731)

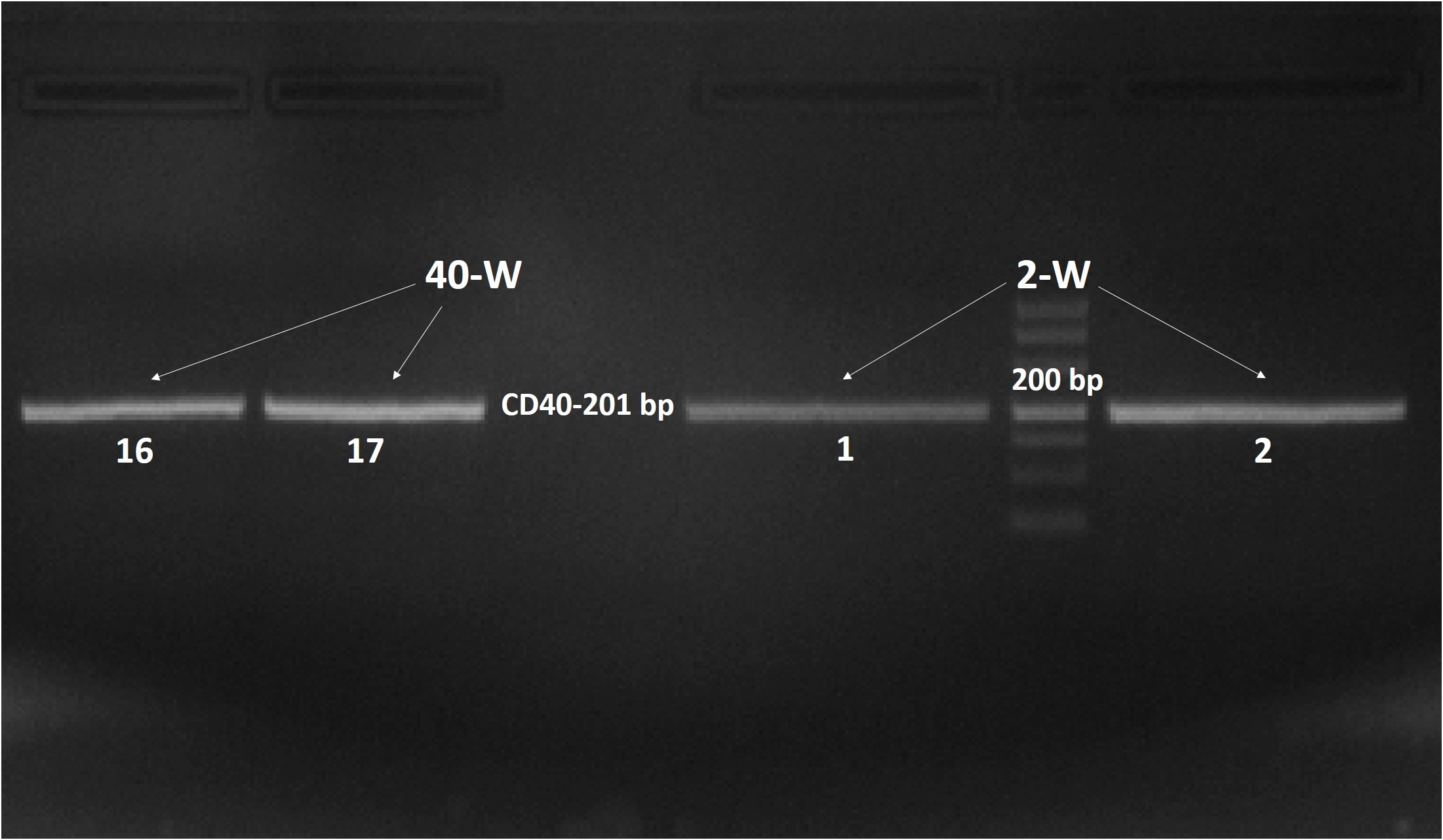

Supplement: Figure S1 — Agrose gel electrophoresis of BSP product. The BSP product showed a clear band in 2% agarose gels, and it contained about 200 bases. [file Image_1.JPEG]

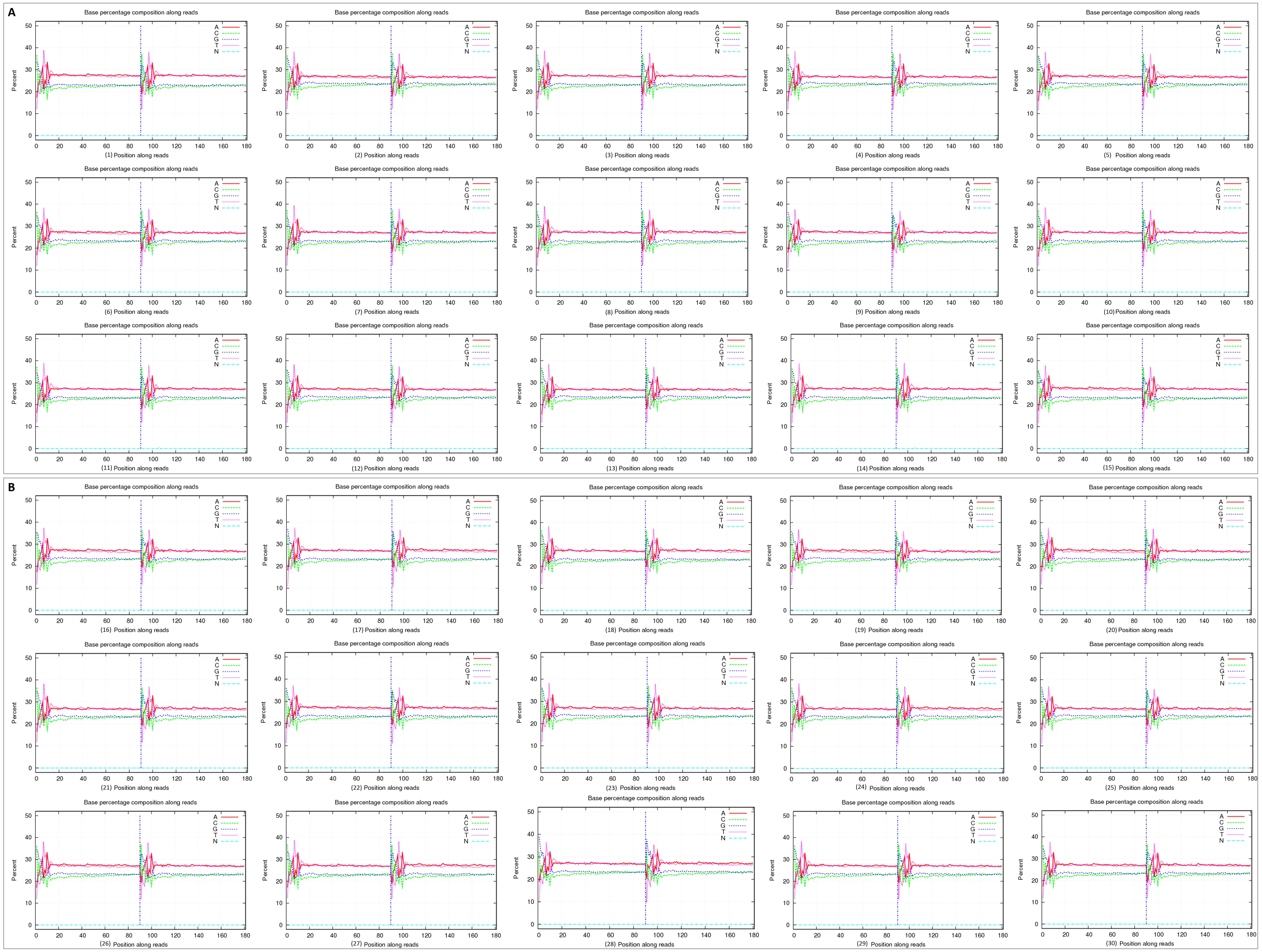

Supplement: Figure S2 — The base percentage composition along raw reads. [file Image_2.JPEG]

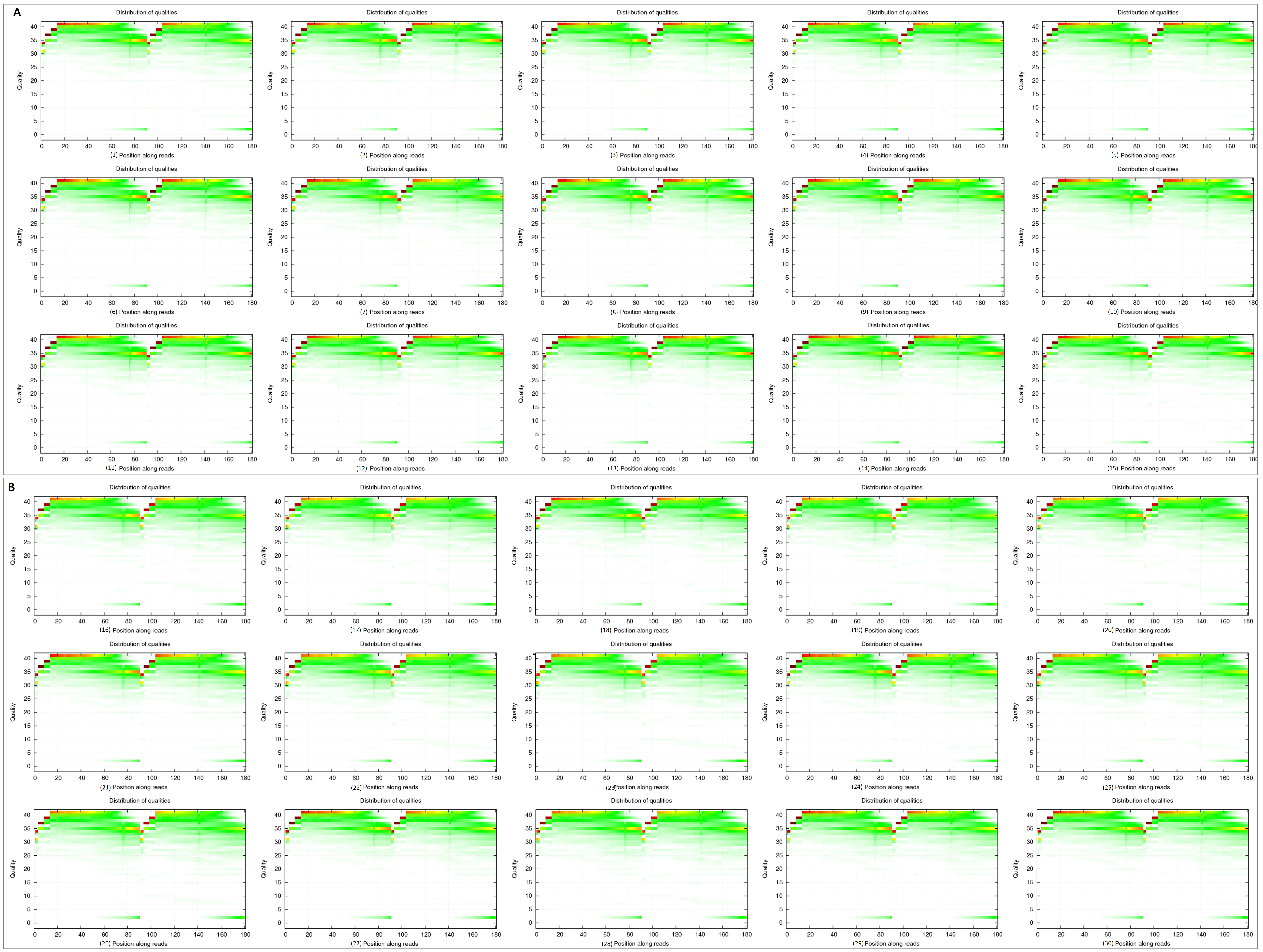

Supplement: Figure S3 — The quality distribution of bases along reads. [file Image_3.JPEG]

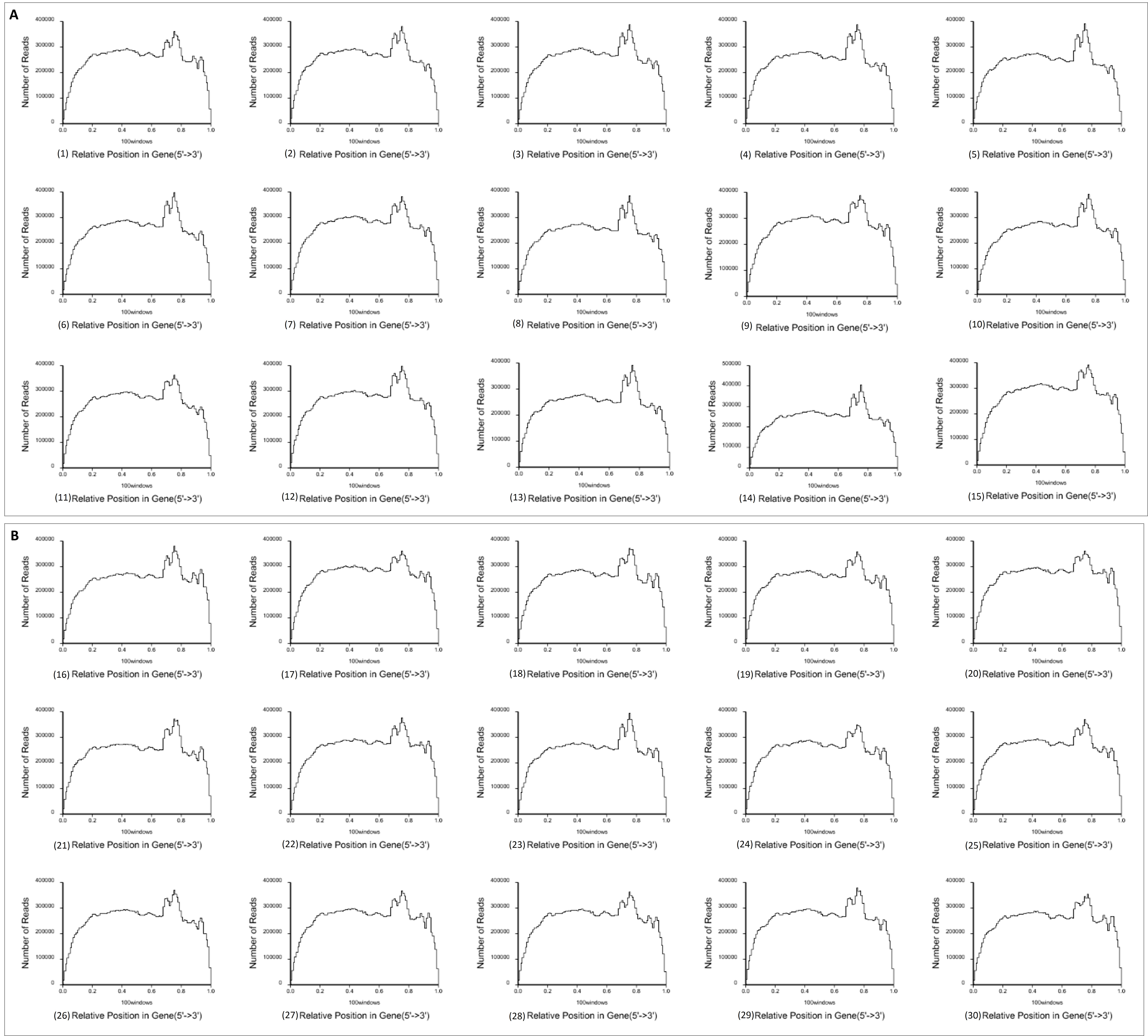

Supplement: Figure S4 — Randomness assessment of the samples. [file Image_4.JPEG]
